# Tickborne Relapsing Fever Caused by *Borrelia persica*, Uzbekistan and Tajikistan

**DOI:** 10.3201/eid1707.101894

**Published:** 2011-07

**Authors:** Nathalie Colin de Verdière, Samia Hamane, Marc-Victor Assous, Natacha Sertour, Elisabeth Ferquel, Muriel Cornet

**Affiliations:** Author affiliations: Hôpital Saint-Louis, Paris, France (N. Colin de Verdière, S. Hamane);; Shaare Zedek Medical Center, Jerusalem, Israel (M.V. Assous);; Institut Pasteur, Paris (N. Sertour, E. Ferquel, M. Cornet);; Université Joseph Fourier, Grenoble, France (M. Cornet)

**Keywords:** *Borrelia persica*, tickborne, relapsing fever, diagnosis, molecular identification, bacteria, Uzbekistan, Tajikistan, letter

**To the Editor:** Tickborne relapsing fever (TBRF) is caused by several *Borrelia* species and transmitted by argasid soft ticks of the genus *Ornithodoros*. The disease is endemic to many parts of the world, especially Africa (*B. duttonii* and *B. crocidurae* most prevalent), and the Mediterranean basin (*B. hispanica* most prevalent)*.* In Eurasia, TBRF is mainly caused by *B. persica* (*1*–*3*). We report a patient who returned to France with *B. persica* infection after visiting Uzbekistan and Tajikistan.

In September 2008, a 32-year-old man sought care at the Saint Louis Hospital in Paris, France, for a fifth relapsing episode of fever. Three weeks earlier (July 31–August 18), he had trekked through Uzbekistan and Tajikistan and spent 7 nights in a tent in Uzbekistan. The day before his return to France, he had fever (39.5°C) that lasted 5 days without other symptoms. After a 4-day interval, fever recurred for 1 day, then relapsed 3 other times (every 2 days with fever for 12 hours). Laboratory investigations performed earlier by his general physician on August 19 and 20 showed lymphopenia (0.76 × 10^9^ cells/L and 0.44 × 10^9^ cells/L), thrombocytopenia (94 ×10^9^ cells/L and 80 ×10^9^ cells/L), and C-reactive protein level 300 mg/L (reference level <6 mg/L).

Physical examination when the man was afebrile found no clinical abnormalities. He did not recall tick bites, and no lesion was seen on his skin. Laboratory investigations on September 2 indicated persistant lymphopenia (1.0 × 10^9^ cells/L), C-reactive protein 96 mg/L, and mild cholestasis (alkaline phosphatase level 125 UI/L and gamma glutamyl transferase level 92 UI/L). Thick and thin Giemsa-stained blood smears showed neither *Plasmodium* spp. nor *Borrelia* spp. However, quantitative buffy coat analysis (QBC; Becton Dickinson, Le Pont de Claix, France) showed numerous spirochetes, which prompted a rereview and careful analysis of the slides, during which spirochetes were infrequently seen. PCR and sequencing of the 16S rRNA gene, performed as previously described, from a whole blood sample identified these bacteria as *B. persica* (*1*).

According to current recommendations in France, the patient was given doxycycline, 200 mg/d, for 10 days. During the first 12 hours, he was monitored for a Jarish–Herxheimer reaction, which was not observed. By the end of therapy, inflammatory syndrome and lymphopenia had resolved.

Despite high-level sequence conservation, identification of *Borrelia* spp. by sequencing the 16S rRNA gene is reliable and useful for clinical practice (*1*,*4*–*6*). The sequence obtained from the patient reported here was identical (100% identity over 1,472 bp) to the *B. persica* HM161645 reference sequence available from GenBank and sampled from the Galilee region of Israel. It differed by 2 nt (99.86% identity) from *B. persica* U42297 and another unpublished sequence (*B. persica* 11/95), each from Iran. *B. persica* 11/95 was obtained from blood from a rodent collected in Iran and examined at the Institut Pasteur of Iran. We submitted the 2 *B. persica* sequences (1 from the patient reported here and 1 from the 11/95 sample) to GenBank (accession nos. HQ610930 and HQ610931, respectively). We confirmed identification of *B. persica* from the patient reported here by sequencing of both *fla*B and intergenic spacer domains (data not shown), as described (*7*,*8*).

Although recently reviewed, the epidemiology of TBRF in the area of the former Union of Soviet Socialist Republics has not been extensively described (*3*). In most of these countries, the infection is mainly attributed to *B. persica* and transmitted by *Ornithodoros tholozani* ticks, which live in caves, soil, wall crevices, houses, and cow sheds. Mammalian reservoirs, if any, are not known (*3*). However, other agents have been reported, such as *B. latyschewii* (transmitted *by O. tartakovskyi* ticks, which inhabit rodent burrows) in Kazakhstan, Uzbekistan, and Turkmenistan and *B. caucasica* (transmitted by *O. verrucosus* ticks) in the western shore of the Caspian Sea (Armenia, Azerbaijan, and Georgia) (*9*,*10*). Other soft ticks (*O. nereensis* and *O. alactagalis*) have been described in central Asia.

To our knowledge, TBRF cases caused by *B. persica* in Uzbekistan and Tajikistan have been rarely reported. The clinical illness of the patient reported here did not differ substantially from that of patients in Israel, Iran, or Jordan, where the infection is more frequently detected. The illness appears benign without chills, headache, vomiting, arthralgia, epistaxis, or hematuria. However, lymphopenia, which resolved rapidly, has not been described for other TBRF cases.

Our report highlights that TBRF is endemic to countries of the former Union of Soviet Socialist Republics. Physicians should consider this diagnosis for febrile patient returning from this area. Efforts to prevent tick bites should be emphasized. Accurate microbiological diagnosis comprises molecular detection or quantitative buffy coat analyses, each of which enhances sensitivity. However, in most disease-endemic countries, diagnosis is based only on examination of direct blood smears, which can lead to false-negative results and underestimation of the actual extent of this infection (*1*).
